# Hyperglycemia-induced Renal P2X7 Receptor Activation Enhances Diabetes-related Injury

**DOI:** 10.1016/j.ebiom.2017.04.011

**Published:** 2017-04-20

**Authors:** Robert I. Menzies, John W.R. Booth, John J. Mullins, Matthew A. Bailey, Frederick W.K. Tam, Jill T. Norman, Robert J. Unwin

**Affiliations:** a British Heart Foundation Centre for Cardiovascular Science, The University of Edinburgh, Edinburgh, UK; b UCL Centre for Nephrology, University College London, London, UK; c Imperial College Renal and Transplant Centre, Department of Medicine, Imperial College London, London, UK; d Cardiovascular and Metabolic Diseases (CVMD) iMed, AstraZeneca, Gothenburg, Sweden

**Keywords:** Glucose, Purine, P2, Renal, Cytokine, CKD

## Abstract

Diabetes is a leading cause of renal disease. Glomerular mesangial expansion and fibrosis are hallmarks of diabetic nephropathy and this is thought to be promoted by infiltration of circulating macrophages. Monocyte chemoattractant protein-1 (MCP-1) has been shown to attract macrophages in kidney diseases. P2X7 receptors (P2X7R) are highly expressed on macrophages and are essential components of pro-inflammatory signaling in multiple tissues. Here we show that in diabetic patients, renal P2X7R expression is associated with severe mesangial expansion, impaired glomerular filtration (≤ 40 ml/min/1.73 sq. m.), and increased interstitial fibrosis. P2X7R activation enhanced the release of MCP-1 in human mesangial cells cultured under high glucose conditions. In mice, P2X7R-deficiency prevented glomerular macrophage attraction and collagen IV deposition; however, the more severe interstitial inflammation and fibrosis often seen in human diabetic kidney diseases was not modelled. Finally, we demonstrate that a P2X7R inhibitor (AZ11657312) can reduce renal macrophage accrual following the establishment of hyperglycemia in a model of diabetic nephropathy. Collectively these data suggest that P2X7R activation may contribute to the high prevalence of kidney disease found in diabetics.

## Highlights

• Renal P2X7 receptor (P2X7R) expression is associated with chronic kidney disease and fibrosis in human diabetes.
• Human mesangial P2X7R activates to regulate monocyte chemoattractant release under high glucose conditions.
• Genetic loss of murine P2rx7 gene is associated with reduced renal macrophage accrual and glomerular fibrosis.
• Chronic inhibition of P2X7R limits renal macrophage accumulation in a model of diabetic nephropathy.

The global prevalence of diabetes has doubled in recent decades and is the most common cause of kidney failure. Unfortunately, there are few therapeutic options to halt or limit kidney disease progression. P2X7R has emerged as a potential target in multiple kidney disease models. We have investigated P2X7R expression in diabetic human renal biopsy samples, high-glucose treated cells and P2X7R-deficient mice. Our collective findings indicate that kidney P2X7R expression causes macrophage accrual and fibrosis in diabetic kidneys. As proof of concept, we demonstrate that pharmaceutical inhibition of P2X7R, in a rodent model, prevents the seemingly deleterious accumulation of kidney macrophages.

## 1 Introduction

Diabetic kidney disease is becoming a global epidemic (Gross et al., 2005) and is expected to increase in prevalence as the diabetic population grows (Shaw et al., 2010). Diabetic nephropathy is a unique predictor of mortality in both insulin-dependent and -independent diabetes (Afkarian et al., 2013, Groop et al., 2009). Antihypertensive, antiproteinuric and blood-glucose lowering therapies are the mainstays of treatment. However, despite aggressive blood pressure management (Gross et al., 2005, Parving et al., 1983) and hyperglycemic control (The Diabetes Control and Complications Trial Research Group, 1993), most diabetic patients progress to chronic kidney disease (CKD) and eventually end-stage renal failure.

Diabetic nephropathy is driven by mobilization of major inflammatory cascades (Wada and Makino, 2013) that result in glomerular extracellular matrix (ECM) deposition, basement membrane thickening, mesangial sclerosis, proteinuria and interstitial fibrosis (Mason and Wahab, 2003, Tervaert et al., 2010). Pro-inflammatory signaling may begin in the glomerular mesangium (Gómez-Guerrero et al., 2005, Savill et al., 1992). Exposure to a high glucose *milieu* can enhance mesangial secretion of the monocyte chemoattractant protein-1 (MCP-1) resulting in macrophage accumulation and glomerulonephritis (Ihm et al., 1998, Wada et al., 2000), leading ultimately to fibrosis (Zeisberg and Neilson, 2010). In diabetic patients, the ratio of MCP-1 to creatinine is a predictor of reduced glomerular filtration rate (GFR) and renal impairment (Tam et al., 2009).

Multiple adenosine-5′-triphosphate (ATP)-activated (P2X) receptors and G-protein coupled purinergic (P2Y) receptors regulate MCP-1 secretion in cell models (Shieh et al., 2014, Stokes and Surprenant, 2007). There are seven P2X receptors that when activated by ATP, function as non-selective cation channels (Costa-Junior et al., 2011). The P2X7 receptor (P2X7R) has a uniquely long carboxyl domain that interacts with membrane pores such as pannexin-1 - to drive inflammation, cell death, and tissue remodelling (Lister et al., 2007, Menzies et al., 2015b). P2X7R is highly expressed on macrophages where it regulates the maturation and release of cytokines (Ferrari et al., 2006), and on mesangial cells where it promotes ECM expansion (Solini et al., 2014, Solini et al., 2005). P2X7R is also expressed on non-immune cells, including those of the pancreas (Coutinho-Silva et al., 2007) and renal microvasculature (Menzies et al., 2013).

Preclinical research has identified P2X7R as an attractive therapeutic target in kidney diseases (Menzies et al., 2016, Solini et al., 2013). However, it is not yet clear whether P2X7R activation regulates human diabetic nephropathy or, specifically, if glomerular P2X7R activation underlies disease severity. In the present study P2X7R was immunolocalized in renal biopsies from diabetic patients and assessed against indicators of renal function and disease. Resident glomerular cells facilitate the accrual of macrophages and so P2X7R-mediated proinflammatory signaling was investigated in primary human mesangial cells exposed to high glucose. We next investigated whether P2X7R-deficiency protected against diabetic renal injury in a murine model. In a final experiment, we investigated the therapeutic potential of a P2X7R inhibitor (P2X7Ri) following the establishment of hyperglycemia in a rat model of diabetic nephropathy.

## 2 Materials and Methods

### 2.1 Human Biopsy Samples

Renal biopsy specimens were obtained from patients after informed consent (local ethics committee approval; NRES Committee London - West London & GTAC; 04/Q0406/25), and only materials surplus to clinical need were used. Thin basement membrane (TBM) biopsies served as controls for diabetic nephropathy. TBM represents the carrier state for autosomal recessive Alport Syndrome, in which there are mutations in basement membrane collagen typically without renal impairment (Savige et al., 2013). Paraffin sections were retrieved from the histology archive at Hammersmith Hospital for 3 TBM patients and 30 diabetics (19/30; 63% type II).

### 2.2 Immunohistochemistry

Primary sections were de-waxed through xylenes and graded alcohols to water. Heat-induced epitope retrieval (HIER) was used. Sections were placed in boiling sodium citrate buffer (0.01 M; pH 6.0) and transferred to a water bath (95 °C for 20 min). Sections were then cooled under running water for 5 min and placed in phosphate-buffered saline (PBS; Sigma-Aldrich, Poole, UK). Sections were incubated in 0.3% hydrogen peroxide in PBS (10 min) then washed in PBS 3 times for 5 min each. Sections were incubated in either 10% dry powdered milk (Marvel; Premier Foods, St Albans, UK) or 20% normal goat serum (Dako; Carpinteria, CA, USA) in PBS for 30 min at room temperature.

Primary antibodies for P2X7R (APR-004, Alomone, RRID: AB_2040068; 1:50 murine; 1:100 human), human CD68 (Clone KP-1, Dako, RRID: AB_563621; 1:100), murine CD68 (Clone FA-11, Serotec, RRID: AB_323909; 1:200) and collagen IV (Ab6586, Abcam, RRID: AB_305584; 1:100) were reconstituted in PBS. Blocking solution was tapped off the slide and residual solution blotted. Primary antibody was applied at sufficient volume to cover the section (typically 100–200 μl). Slides were incubated in a covered, humidified staining chamber for 1 h (or overnight for human P2X7R staining). Negative controls comprised omission of primary antibody, addition of polyclonal normal rabbit immunoglobulin at an equivalent concentration to the primary antibody, and use of primary antibody pre-incubated with the cognate immunizing peptide at a 1:1 ratio. Detection was performed by a commercial system (EnVision; Dako, Carpinteria, CA, USA). Positive staining appeared as brown deposits.

Glomerular macrophage (CD68 +) number was counted for 20 glomeruli in each section and the mean value obtained. The same starting point was used in each section and the first 20 randomly encountered glomeruli were included. Interstitial macrophage number was counted for 5 randomly selected fields of view (× 200 magnification) and the mean value obtained. Glomeruli were outlined and area measured and type IV collagen calculated as percentage stained area per glomerular area. Fifteen glomeruli in each section were scored and the mean value taken.

### 2.3 Primary Human Mesangial Cells

Primary human mesangial cells (pHMCs; Lonza Biologics, Slough, UK) were cultured in a proprietary basal medium supplemented with 5% fetal calf serum (FCS) and gentamicin amphotericin-B (Sigma-Aldrich, Poole, UK). Cells were grown at 37 °C in a humidified atmosphere of 5% CO_2_. Medium was changed every 2–3 days. At 90–100% confluence cells were sub-cultured onto chamber slides (Nunc, Lab-Tek, Thermo Fisher Scientific, Waltham, MA, USA) or 96-well plates (Nunc, Maxisorp, Thermo Fisher Scientific). Viability of pHMCs cultured in the presence of small molecule receptor inhibitors was assessed using a 3-(4,5-dimethylthiazol-2-yl)-5-(3-carboxymethoxyphenyl)-2-(4-sulfophenyl)-2H-tetrazolium, inner salt (MTS) assay (CellTiter 96; Promega, Madison, WI, USA).

For experiments cells were grown > 70% confluence and then made quiescent by incubation in glucose-free RPMI 1640 supplemented with 4 mM D-glucose with no serum for 24 h to induce cell cycle synchronization and quiescence prior to treatment. All experiments were done in serum free medium. Supplementing glucose-free RPMI 1640 with 30 mM D-glucose simulated hyperglycemic conditions. L-glucose, the metabolically inactive stereoisomer of D-glucose, was added to 4 mM D-glucose media at a concentration of 26 mM to act as an osmotic control for hyperglycemic media. Cells were grown in these conditions for 48 h without a medium change, before collection of supernatants for MCP-1 quantification (below) or cell monolayer lysates for protein extraction.

The effects of a selective P2X7R inhibitor (A438079; http://www.guidetopharmacology.org/GRAC/LigandDisplayForward?tab=biology&ligandId=4118; Tocris Bioscience, Bristol, UK), a P2X7R agonist BzATP, (Tocris), on MCP-1 production were tested both in the presence and absence of hyperglycemia by supplementing 4 mM D-glucose and 30 mM D-glucose media respectively, with the appropriate reagent concentration. Cells were treated for 30 min before collecting supernatants.

### 2.4 Protein Extraction

Cells were washed twice with ice-cold PBS before adding 100 μl of a proprietary cell lysis buffer (Invitrogen; Constituents: 10 mM Tris pH 7.4, 100 mM NaCl, 1 mM EDTA, 1 mM EGTA, 1 mM NaF, 20 mM Na4P2O7, 2 mM Na3VO4, 1% Triton X-100, 10% glycerol, 0.1% SDS, 0.5% deoxycholate) supplemented with 1 mM phenylmethanesulfonylfluoride (PMSF; a serine protease inhibitor) and protease inhibitor cocktail (P2714, Sigma Aldrich, Poole, UK). Monolayers were agitated for 2–3 min before scraping and collecting the lysate into microcentrifuge tubes. Tubes were rested on ice for 30 min, vortexing at 10 minute intervals, to ensure complete lysis of cell membranes and solubilization of proteins. The extract was then centrifuged at 18,000 ×* g* for 10 min at 4^o^C to pellet insoluble cellular debris. Supernatant was transferred to a fresh microcentrifuge tube and protein concentration quantified (Pierce BCA kit, Thermo Fisher Scientific).

### 2.5 Immunofluorescent Staining of P2X7R in pHMC

Medium was removed and cells washed twice with PBS (Sigma-Aldrich, Poole, UK). Cells were fixed in 4% paraformaldehyde (PFA; Sigma-Aldrich, Poole, UK) in PBS for 5 min at room temperature and washed 3 times with PBS. Cells were permeabilized with 1 ml 0.1% Triton X-100 (Sigma-Aldrich) in PBS for 10 min and washed twice with PBS.

Non-specific binding sites were blocked by incubation with 5% BSA in 0.1% Triton in PBS for 1 h at room temperature. Primary P2X7R antibody (Ab109246; Abcam, RRID: AB_10858498, Cambridge, UK) or control (polyclonal IgG or diluent alone) diluted 1:2000 in 10% Marvel in PBS (Marvel; Premier Foods, St Albans, UK) was applied overnight. Cells were then washed 3 times in PBS for 5 min each and incubated with a fluorochrome-conjugated secondary antibody (Alexa 488; Invitrogen, Paisley, UK) diluted in 5% BSA in 0.1% Triton in PBS for 1 h in the dark. Cells were washed with PBS, nuclei visualized with DAPI (Vector Laboratories, Peterborough, UK).

### 2.6 Quantification of pHMC MCP-1

A 96-well plate (Nunc ‘Maxisorp’, Thermo Fisher Scientific) was coated with 100 μl of an affinity-purified mouse anti-human MCP-1 antibody (R&D Systems, Abingdon, UK) at a concentration of 2 μg/ml diluted in PBS. The plate was covered with an adhesive strip (Appleton Woods, Birmingham, UK) and placed at 4^o^C overnight to allow adsorption of antibody to the walls of the microplate. Recombinant human MCP-1 (R&D Systems) was used to create a reference concentration series to form a 9-point standard series (9.76–2500 pg/ml). A ‘blank’ sample comprising diluent alone was also included. Following washing with buffer, an affinity-purified biotinylated polyclonal goat anti-human MCP-1 antibody (100 ng/ml; R&D systems, Minneapolis, MN, USA) was added and signal amplification performed with streptavidin-HRP conjugate. MCP-1 intensity was measured by absorbance (450 nm) in the presence of 3,3′,5,5′-tetramethylbenzidine (TMB; Cambridge Bioscience, Cambridge, UK) in an ELx800 spectrophotometric microplate reader (BioTek, Winooski, VT, USA).

### 2.7 Animals

Home Office Science Unit licensed all animal procedures under the Animals (Scientific Procedures) Act 1986. Mice and rats were maintained in a pathogen-free animal facility at Imperial College London (Hammersmith Hospital campus) in individually ventilated cages with free access to water and standard laboratory diet. Maximum cage occupancy was five mice per cage. All experimental animals were male. Gene targeted or littermate control mice were on the C57BL/6 background. P2X7R-deficient mice (Ke et al., 2003) were bred in-house from heterozygote breeding pairs provided by GlaxoSmithKline (GSK, Brentford, UK); with littermate, wild-type (WT) animals used as experimental controls. Notably this is a “knock-down” model in which the protein coding murine P2X7k alternative transcript is expressed (Nicke et al., 2009). Indeed, background P2X7R mRNA signal was detected in P2X7R-deficient mice, attributed to primer binding of these alternative transcripts. Genotype PCR was performed using oligonucleotide primers designed to discriminate between native (5′-3′: TGCCCATCTTCTGAACACC; 5′-3′: CTTCCTCTTACTGTTTCCTCCC) and disrupted (5′-3′: TGCCCATCTTCTGAACACC; 5′-3′: GCAAGGCGATTAAGTTGGG) forms of the P2rx7 gene.

### 2.8 Type 1 Diabetic Murine Model

WT and P2X7R-deficient mice aged 8 months or older were randomly divided into two groups: receiving either streptozotocin (STZ; Sigma-Aldrich, Poole, UK) 50 mg/kg IP dissolved in 100 mM sodium citrate buffer at pH 4.5 (n = 8), or citrate buffer alone (n = 8). STZ injections were performed on 5 consecutive days, initially after a 4–6 hour fast (DiaComp repetitive low-dose protocol (mouse)). Blood glucose (BG) was measured 3 weeks after the last injection. Mice were immobilized in a restrainer, a tail vein ‘prick’ performed with a 23G needle and a OneTouch glucometer (Lifescan, Milpitas, CA, USA) used for glucose measurement. BG > 16 mmol/l was regarded as diabetic. BG was measured at additional 3 weekly intervals during the study, and animals with readings falling outside of the desired range were excluded. Urine was collected at baseline and week 12; terminal collection of blood and kidney tissues was performed at week 12.

### 2.9 Serum and Biochemical Analysis

The concentration of urinary albumin in mice was measured using a specific, indirect sandwich ELISA (Bethyl Laboratories, Montgomery, TX, USA). Urinary albumin concentration values (transformed from ODs using the standard curve and expressed in mg/l) were divided by paired urinary creatinine to give the urine albumin to creatinine ratio (uACR; mg/mmol creatinine). Urinary creatinine was measured using the Jaffe reaction. Alkaline picrate was produced by adding 2.5 ml of 1 N sodium hydroxide to 12.5 ml of picric acid against standards (0.03125–2 mmol/l) in duplicate and measured at 490 nm (ELx800; BioTek, Potton, UK). Serum creatinine was measured from whole blood supernatant (centrifugation at 240 ×* g* for 5 min). Creatinine was measured by the modified Jaffe reaction in an Olympus AU600 analyzer (Olympus, Watford, UK) by the clinical biochemistry laboratory at the Hammersmith Hospital (London, UK).

### 2.10 Quantification of Tissue Histology

Glomerular size was measured on periodic acid-Schiff (PAS)-stained sections. Working systematically from one corner of the tissue section, the first 30 glomeruli encountered were digitized using a scientific grade digital color camera (Olympus, Tokyo, Japan) at 400 × magnification. Scale was calibrated using an etched graticule of known diameter. Subsequent image analysis was performed using ImagePro software (Media Cybernetics, Bethesda, MD, USA). Glomeruli were carefully outlined and each glomerular tuft isolated for surface area calculation. At least 30 glomerular areas were calculated from each animal.

Tissue injury was assessed on Picrosirius red-stained sections to detect collagen under polarized light. For each section, 5 randomly selected cortical fields were photographed and digitized at 200 × magnification. Blood vessels larger than neighboring tubules were systematically excluded from selected fields to prevent skewing of results due to the collagenous component of vessel walls. Using ImagePro, images were converted to gray-scale, rendering birefringent collagen fibrils light gray or white. Picrosirius red-stained areas were highlighted automatically. The highlighted area could then be measured and expressed as a fraction of the area of the section. The mean value for 5 cortical fields of view was calculated for each animal.

### 2.11 Rat Uninephrectomy

Right uninephrectomy (UNx; n = 25) or sham surgery (Sham; n = 8) was performed on male Wistar Han IGS rats (Crl:WI (Han); Charles River; Margate, UK) weighing 200–250 g. Rats were anaesthetised with a 1.5–2% isoflurane oxygen mix containing 0.5 l/min air. The right flank was shaved and a small incision made to expose the kidney. The adipose capsule of the kidney was removed to provide access for a cross-clamp placed over the vascular pedicle and ureter. A silk tie was used to ligate the vessels and ureter proximal to the clamp and the kidney removed. The clamp was removed and the muscle layer sutured closed using an absorbable stitch in a continuous pattern; 3–4 clips were applied to the skin. A second group of rats underwent sham nephrectomy following the procedure described above but with both kidneys left in situ. Rats received a single subcutaneous dose of buprenorphine analgesia (0.02 mg/kg) at the end of the operation. Enrofloxacin (Baytril; Bayer, Leverkusen, Germany) was added to drinking water for 48 h post-operatively as antibiotic prophylaxis against wound infection.

### 2.12 Induction and Maintenance Streptozotocin-induced Diabetes in Rats

Two weeks after surgery, rats were randomly selected to receive a single intravenous (IV) injection of STZ (40 mg/kg) or citrate buffer vehicle. STZ was dissolved in 100 mmol/l citrate buffer (pH 4.5) at the point of use. Animals were placed under light anaesthesia with isoflurane 1.5–2% and STZ or citrate vehicle injected IV via the dorsal penile vein. Water in drinking bottles was replaced with 10% sucrose for 48 h after injection to combat the risk of early hypoglycaemia due to insulin release from injured beta cells.

Venous blood glucose (BG) was checked at 1 week post-injection using a OneTouch glucometer (Lifescan, Milpitas, CA, USA). Light anaesthesia was induced with isofluorane and a tail vein prick performed with a 23G needle. Animals with a blood glucose of > 33.3 mmol/l were administered slow-release subcutaneous insulin pellets (Linshin, Toronto, Canada) to prevent ketoacidosis. 1/8 insulin pellet provides approximately 0.25 units insulin per day. BG was retested 1 week after insertion and a further 1/8 pellet inserted if BG was persistently > 33.3 mmol/l. This cycle was continued until BG had been titrated equivalently amongst experimental rats.

### 2.13 Administration of a Highly Selective P2X7R Inhibitor to Diabetic Rats

Following the establishment of diabetes at week 8, rats were randomly allocated to receive either drug vehicle (UNx STZ Veh; n = 8), or P2X7R inhibitor (P2X7Ri) at either 10 mg/kg (n = 8) or 50 mg/kg (n = 9). The doses of the P2X7Ri (AZ11657312; www.guidetopharmacology.org/GRAC/LigandDisplayForward?ligandId=7722) were based on previous *in vivo* findings (Broom et al., 2008). AZ11657312 was dissolved in a vehicle of 5% DMSO 95% 2-hydroxypropyl-beta-cyclodextrin (HPBCD) solution. HPBCD was reconstituted as a 20% solution in distilled water. Injection volumes for vehicle-treated animals were calculated to ensure weight-based equivalence to both treatment groups. P2X7Ri or vehicle injections were administered IP twice daily for 4 weeks. Urine collections were made at baseline and 12 weeks in metabolic cages. Following the final urine collection, rats were placed under terminal isoflurane anaesthetic and tissues collected post exsanguination.

### 2.14 Statistics

All values described in the text and figures are expressed as mean ± standard error unless otherwise stated. Statistical comparisons were carried out in GraphPad Prism (GraphPad Software, San Diego, CA, USA). Numerical data comparing two groups were analyzed by the Student's *t*-test for parametric data and Mann-Whitney *U* test for non-parametric data. Categorical data comparing two groups were analyzed with Fisher's exact test. Data comparing more than two groups were analyzed by one-way ANOVA with post-tests corrected by the Bonferroni method for multiple comparisons for parametric data, and Kruskal-Wallis with Dunn's post-test for non-parametric data. P < 0.05 was considered significant.

## 3 Results

### 3.1 Renal P2X7R Expression is Associated With CKD and Fibrosis in Diabetes

**Fig. 1:**
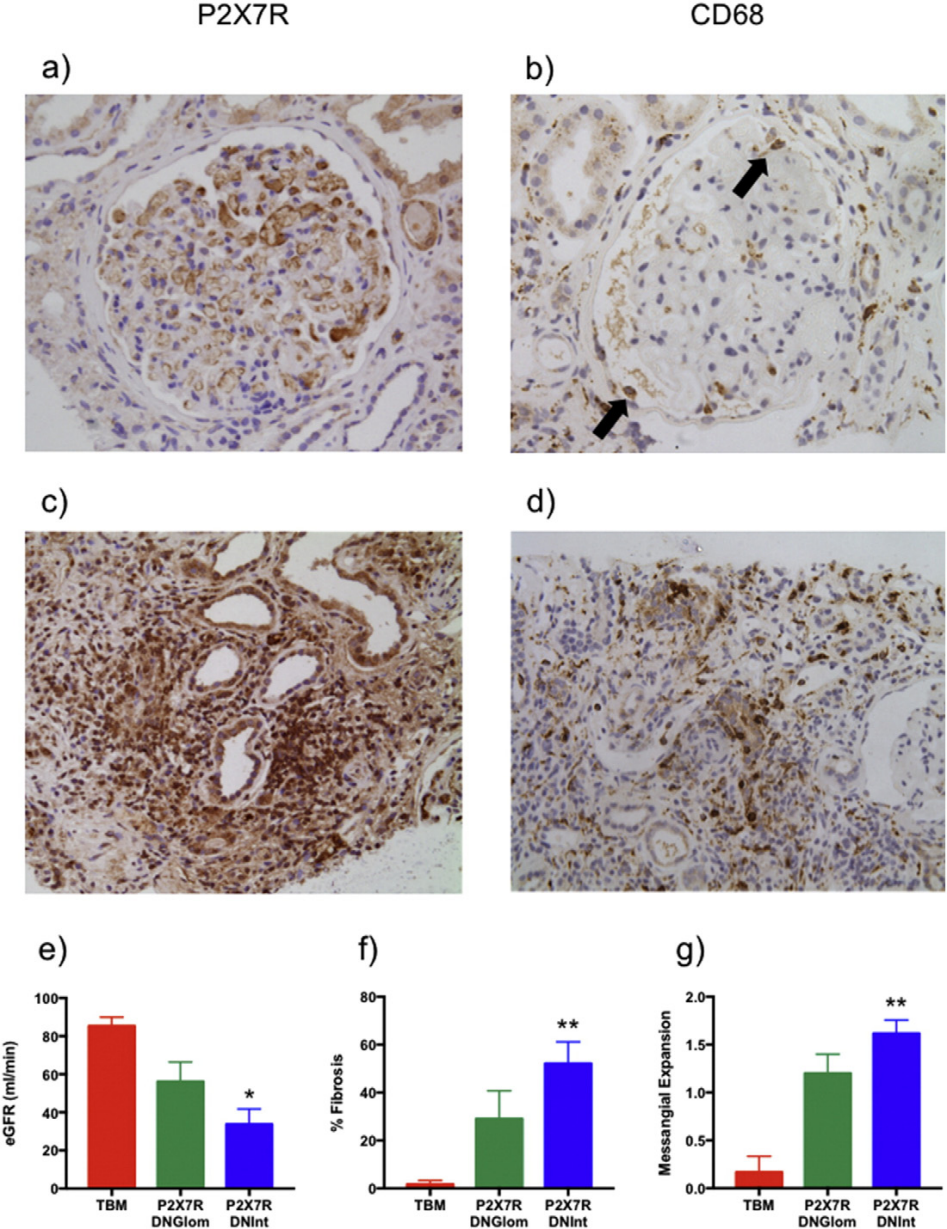
Renal P2X7R in human diabetic nephropathy. Patient biopsies expressing (a) P2X7R protein and (b) CD68 + monocytes in glomeruli indicated with black arrows. In the most severe cases (c) P2X7R staining and (d) CD68 + staining of the interstitium overlapped in regions of inflammation. Estimated glomerular filtration rate (eGFR) (e), fibrosis (f) and mesangial expansion (g) in patients with interstitial (DNInt = 7), glomerular (DNGlom = 5) P2X7R immunoreactivity compared to thin basement membrane (TBM = 3) controls subjects. *P < 0.05, **P < 0.01 DNint vs. TBM. a, b × 200 mag c, d × 100 mag.

In renal biopsy sections from diabetic patients, P2X7R immunolocalized to glomeruli (16/30; 53% patients), either as granular circumferential endothelial deposits in capillary loops or more focally within the glomerular mesangium (Fig. 1). The intensity of glomerular P2X7R staining varied between glomeruli and interstitial compartments (Suppl. Fig. 1a-c). The renal tubules stained positively for P2X7R across the majority (27/30; 90%) of patients. In contrast, no glomerular or interstitial P2X7R staining was observed in biopsies from patients with TBM disease, which served as non-diabetic controls (Suppl. Fig. 2a). Marked P2X7R expression was seen in a subset of tubular epithelial cells with characteristics of the distal tubule (thin cuboidal epithelium with little brush border and open lumens), while minimal proximal tubular staining was seen (Suppl. Fig. 2b). However, non-diabetic P2X7R-positive glomerular staining does occur in other human renal diseases, as indicated in the example of Class IV proliferative lupus nephritis (Suppl. Fig. 2c).

Immunohistochemistry for the macrophage marker CD68 was performed on two patient biopsies, one with significant glomerular P2X7R immunoreactivity and the other with predominantly tubulointerstitial P2X7R expression (Fig. 1b). P2X7R staining appeared more widespread than CD68, including endothelial glomerular structures. Most patients (23/30; 77%) also had evidence of P2X7R-positive staining within the interstitial compartment (Fig. 1c) and in areas of established tubular atrophy where a significant number of infiltrating inflammatory cells were apparent (Fig. 1d). Glomerular and interstitial P2X7R staining was not mutually exclusive, co-existing in 11/30 or 37% of patients.

Renal function was assessed in TBM and a sub-set of the 23 diabetic patients with distinct P2X7R interstitial or glomerular localization. Diabetic patients with only renal interstitial P2X7R staining (DNInt) had significantly lower estimated glomerular filtration rate (eGFR) compared with either patients with only glomerular P2X7R staining (DNGlom) or with TBM control patients (Fig. 1e). Interstitial P2X7R staining was also associated with more severe mesangial expansion in patients with interstitial P2X7R staining with approximately half of the tissue in this compartment undergoing interstitial fibrosis (Fig. 1f); significant expansion of the glomerular mesangium was also detected in this group (Fig. 1g).

### 3.2 Glomerular Mesangial P2X7R Activation Enhances Monocyte Chemoattraction

**Fig. 2:**
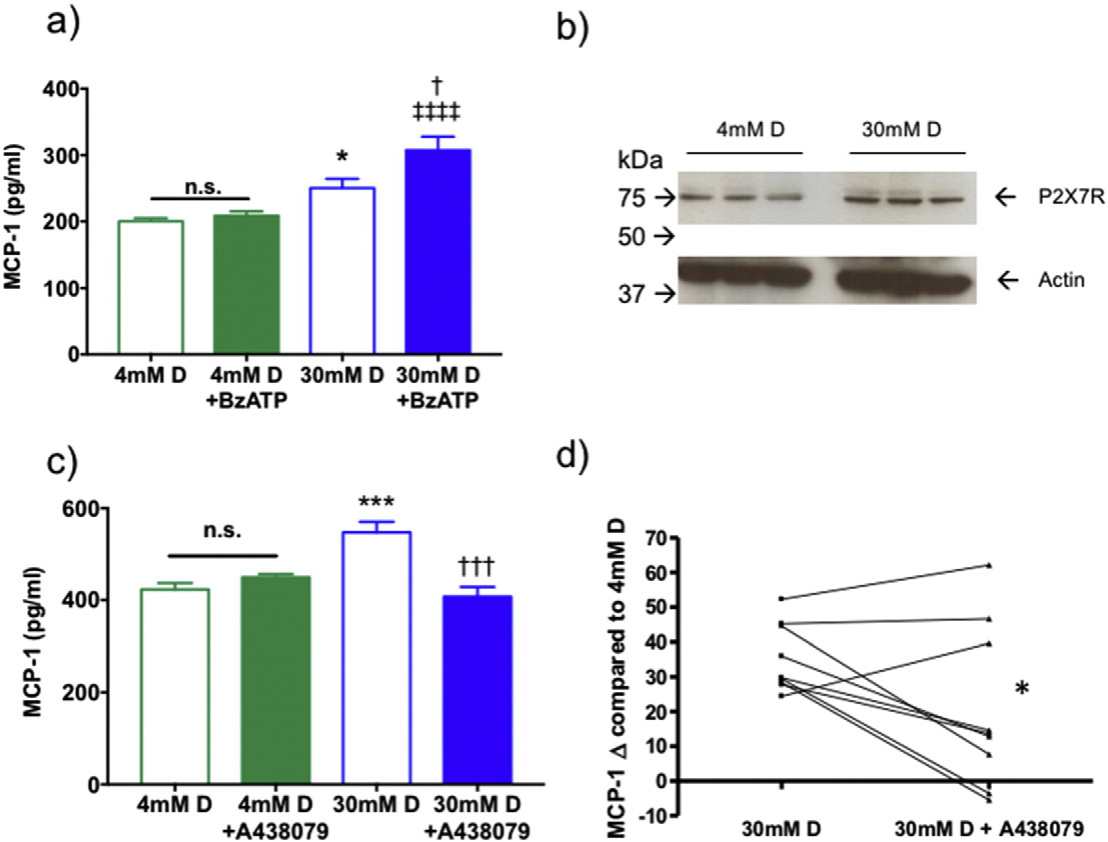
Glomerular mesangial P2X7R and MCP-1. Activation of P2X7R with BzATP increased MCP-1 secretion from pHMCs under high glucose conditions (a). P2X7R protein expression in mesangial cells under normal and high glucose conditions (b). Effect of a P2X7R inhibitor (A438079) on MCP-1 secretion (c). Subtraction of normal glucose MCP-1 release highlighted the absolute magnitude of MCP-1 reduction by P2X7R blockade alone across 9 separate experiments (d). *P < 0.05, **P < 0.01, ***P < 0.001 vs baseline; †P < 0.05, ††P < 0.01, †††P < 0.01 versus 30 mM D pHMC; ‡‡‡‡P < 0.0001, 4 mM D + BzATP vs. 30 mM D + BzATP.

Glomerular mesangial cells attract circulating monocytes through the release of chemoattractants such as MCP-1. Primary human mesangial cells (pHMC's) were used to determine the role of P2X7R-mediated MCP-1 release under high glucose conditions. In controls (4 mmol/l D-glucose), basal MCP-1 levels were not affected by addition of the P2X7R agonist BzATP (Fig. 2a). Exposure of these cells to high glucose (30 mmol/l D-glucose) induced a significant increase in MCP-1 release (P < 0.05) and this effect was greatly amplified by P2X7R activation (P < 0.0001). Notably, 30 mmol/l D-glucose did not change total P2X7R expression in pHMC (Fig. 2b).

The P2X7R inhibitor A438079 completely prevented BzATP and high glucose-induced secretion of MCP-1 (Fig. 2c), with no dose-dependence observed over the range 5-50 μM, (Suppl. Fig. 3). Importantly, A438079 did not impair cell viability (Suppl. Fig. 4). Comparison of nine independent experiments with normalization to baseline glucose levels demonstrated a reproducible reduction in MCP-1 by A438079 (P < 0.05) (Fig. 2d).

### 3.3 P2X7R-deficiency Ameliorates Glomerular Damage in a Type I Diabetic Mouse Model

Type I diabetes mellitus (T1DM) was induced in WT and P2X7R-deficient mice by injection of STZ. Three weeks after the final injection, both genotypes had developed similar levels of hyperglycemia, which also maintained at 12 weeks (Fig. 2a). Plasma creatinine levels increased in both groups following STZ treatment (Suppl. Fig. 5a). Urinary albumin-creatinine levels were unchanged by induction of T1DM (Suppl. Fig. 5b).

**Fig. 3:**
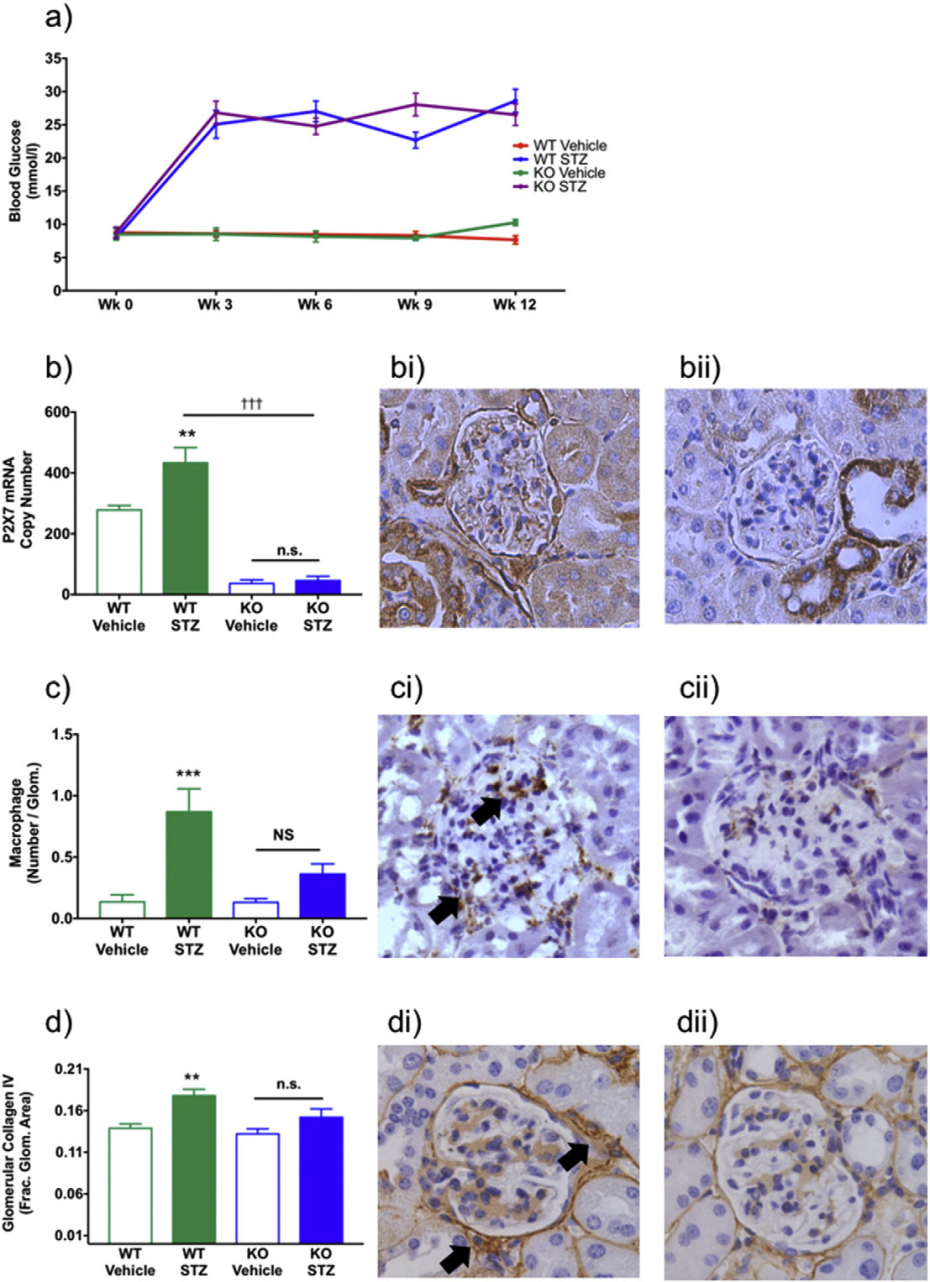
P2X7R-deficient mice and STZ-induced glomerular macrophage accrual. Change in blood glucose following STZ injections (a). Renal P2X7 mRNA levels (b). Glomerular P2X7R staining in WT (bi) and P2X7R-deficient mice (bii). Glomerular macrophage accumulation following STZ (c). Glomerular CD68 staining in WT (ci) and P2X7R-deficient mice (cii). Glomerular type IV collagen deposition increased following STZ (d). Immunolocalization of lomerular collagen IV staining in WT (di) and P2X7R-deficient mice (dii). *P < 0.05, **P < 0.01, ***P < 0.001. WT: wild-type. STZ: streptozotocin. KO: P2X7R-deficient mice. b–d × 200 mag.

T1DM increased whole kidney P2X7R mRNA expression in WT mice (Fig. 3b) and glomerular P2X7R protein expression was also significantly increased (Fig. 3bi). This was not observed in P2X7R-deficient mice (Fig. 3bii). Tubular P2X7R-positive immunoreactivity was observed in all sections, presumed to represent expression of an alternative splice variant (P2X7Rk) in otherwise P2X7R-deficient mice. Distinct interstitial (non-tubular) staining was not observed. CD68-stained macrophages were increased in the glomeruli of diabetic wild-type mice and macrophage accumulation was associated with increased deposition of collagen. In contrast, P2X7R-deficient mice were protected from the diabetes-induced rise in glomerular macrophage number (Fig. 3c) and there was no increase in collagen IV expression. This protection was evident in CD68 stained glomeruli from WT (Fig. 3ci) and P2X7R-deficient mice (Fig. 3cii). Glomerular macrophage accumulation was accompanied by increased collagen IV deposition in WT mice (Fig. 3d, di), which was not observed in P2X7R-deficient mice (Fig. 3d, dii).

### 3.4 P2X7R Inhibition Ameliorates Macrophage Accrual in a Model of Diabetic Nephropathy

Physical and biochemical data at baseline and week 12 are summarized in Table 1. Body weights were similar in all groups at the beginning of the experiment, while diabetic rats gained approximately 25% of the weight gained by control groups over the same period. Kidney weight:body weight ratio more than doubled in UNx STZ Veh rats compared with UNx Veh controls (P < 0.001) and increased by ~ 30% compared with sham-operated and UNx controls (P < 0.001). Rats treated with P2X7Ri had no change in kidney weight:body weight ratio.

**Table 1:** Summary of weight gain and baseline blood glucose in rats. Data from control (Sham and UNx Veh), diabetic (UNx STZ Veh) and P2X7Ri groups at doses stated. Measurements were made at 0 and 12 weeks.

	Sham (n = 8)	UNx Veh (n = 8)	UNx STZ Veh (n = 8)	P2X7Ri (10 mg/kg; n = 8)	P2X7Ri (50 mg/kg; n = 9)
Wk 0 Weight (g)	223.8 ± 12.5	218.8 ± 17.2	217.2 ± 13.3	215.3 ± 7.4	224.4 ± 10.3
% weight gain (Wk 0 to Wk12)	84.0 ± 15.8	84.9 ± 18.7	18.5 ± 8.6a,b	23.3 ± 6.1a,b	25.0 ± 10.8a,b
Wk 0 BG (mmol/l)	4.8 ± 0.8	5.8 ± 0.8	5.1 ± 0.6	5.7 ± 1.5	6.0 ± 1.1
Kidney weight: body weight (mg/kg)	2.70 ± 0.29	3.95 ± 0.23^c^	8.17 ± 0.85a,b	7.88 ± 0.89a,b	7.24 ± 0.98a,b

a,b P < 0.001 vs Sham or UNx Veh.

c P < 0.01 vs Sham.

**Fig. 4:**
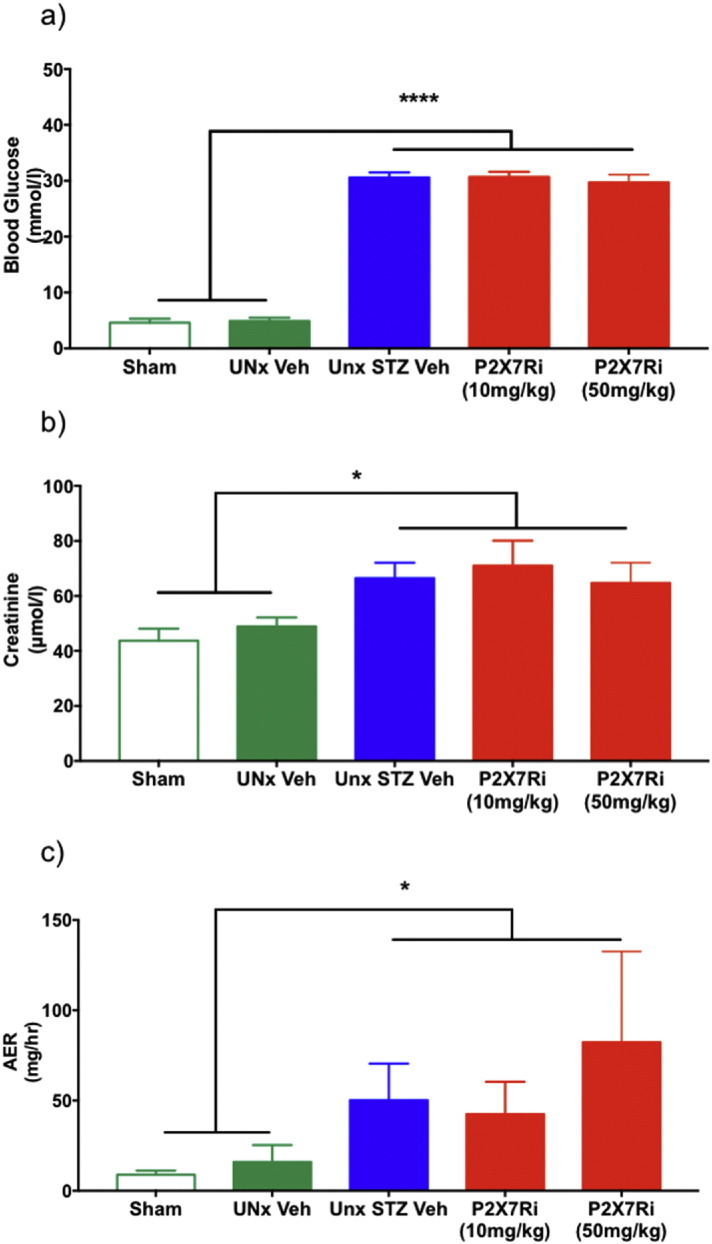
Effect of P2X7R inhibitor on blood glucose levels and kidney function. Blood glucose measured at week 12 (a). Serum creatinine concentration in terminal serum (b) and albumin excretion rate (AER) (c). *P < 0.05 UNx STZ groups vs. Sham or UNx Veh. ****P < 0.0001 UNx STZ groups vs. Sham or UNx Veh. Measurements were made at 12 weeks.

BG was similar across all groups at the beginning of the experiment (Table 1). Hyperglycemia was stably maintained in diabetic animals by insertion of insulin pellets such that hyperglycemia was tightly controlled within the UNx STZ groups (Fig. 4a). Hyperglycemic levels were unaffected by P2X7Ri at either dose. This finding is consistent with observations in P2X7R-deficient mice where STZ injection caused hyperglycemia. Both terminal plasma creatinine and albumin excretion rate (AER) were significantly increased in the diabetic groups (Fig. 4b, c). P2X7Ri did not reduce creatinine levels or albuminuria.

**Fig. 5:**
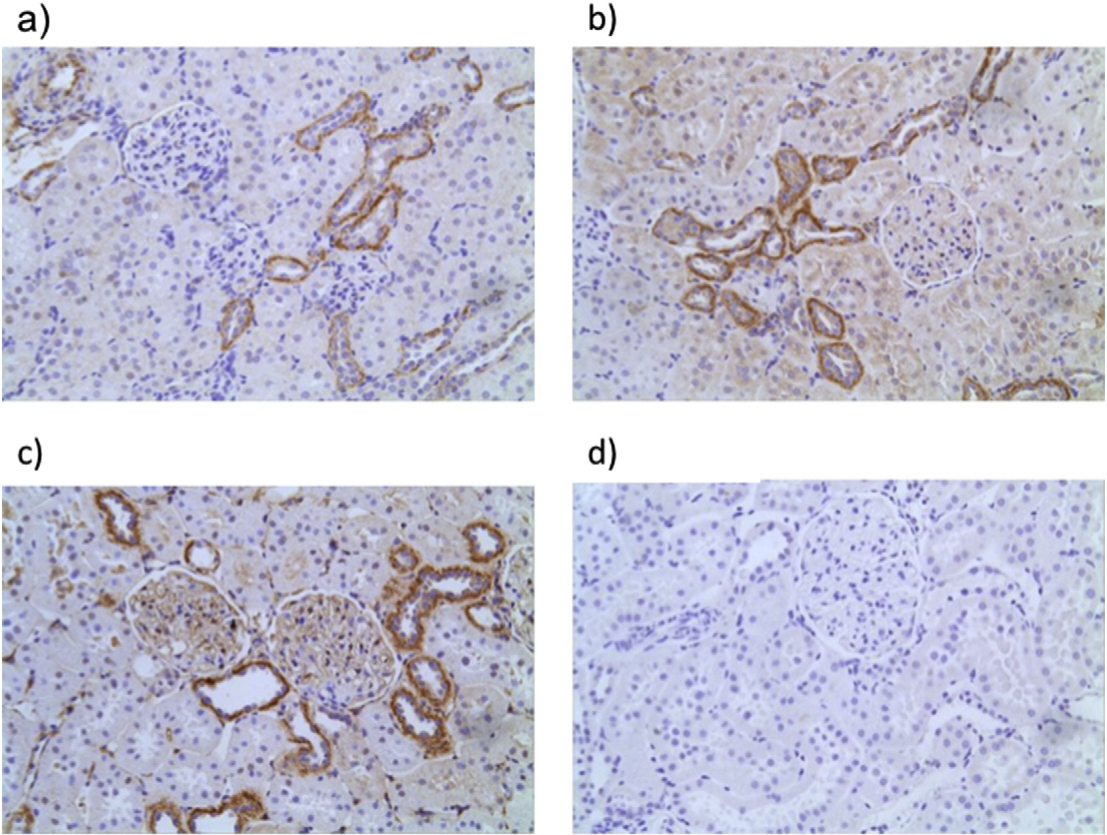
Immunolocalization of P2X7R in rat kidney. Normal Wistar Han kidney collected during UNx (a). Kidney tissue from a week 12 vehicle-injected UNx Wistar Han rat (b). Representative P2X7R staining in a UNx STZ Veh rat kidney at week 12 (c). P2X7R staining in and STZ injected UNx rat where anti-P2X7 antibody was pre-absorbed with its conjugate peptide (1:1) to demonstrate antibody specificity (d). a–d × 200 mag.

P2X7R was immunolocalized in renal sections. In control kidneys removed at UNx, marked basolateral staining was observed in a subpopulation of tubules and endothethial cells (Fig. 5a), but no significant glomerular positivity was detected. The remaining kidney was assessed 12 weeks later (UNx Veh) and a similar staining pattern was observed (Fig. 5b). In the UNx STZ Veh group, P2X7R was highly expressed in glomeruli where marked capillary loop staining became visible (Fig. 5c) additional constitutive expression of P2X7R was also detected in the distal convoluted tubule. Antibody epitope specificity was demonstrated in a UNx STZ Veh section (Fig. 5d).

**Fig. 6:**
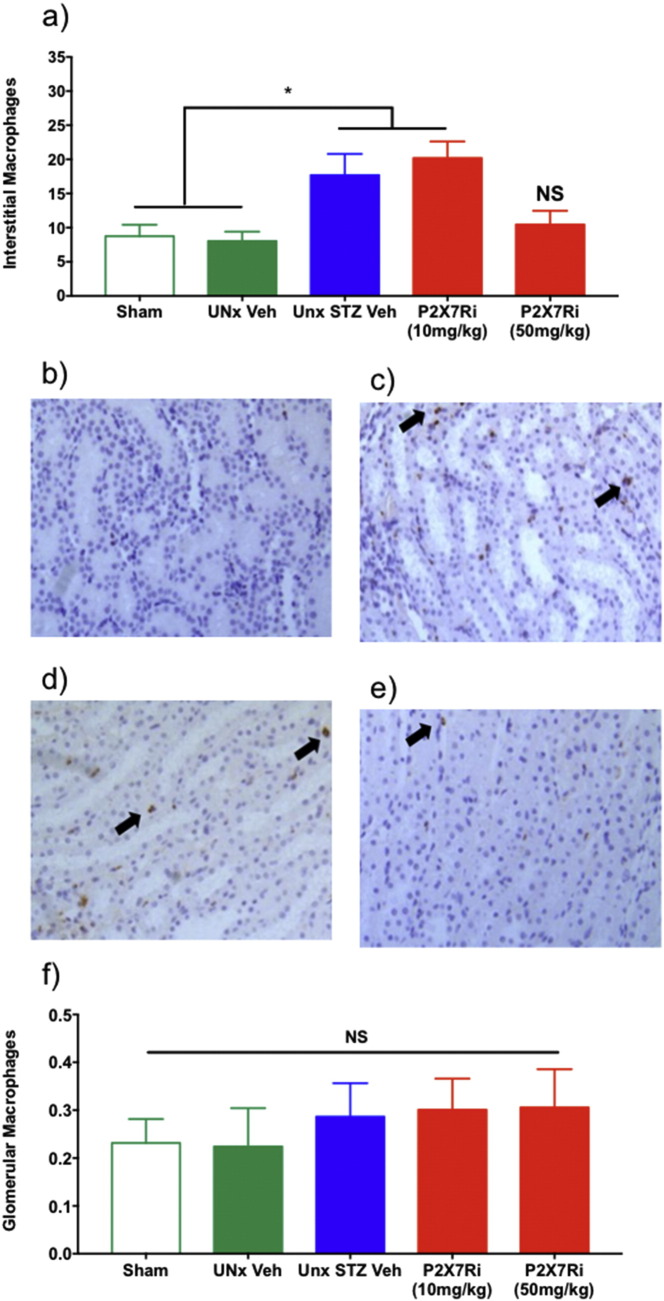
Effect of P2X7R inhibitor on diabetic renal macrophage accrual. Interstitial macrophage abundance (a). Renal interstitial CD68 staining, indicated by black arrows, in sham-treated rat kidney with minimal macrophage staining (b), UNx STZ Drug vehicle-treated diabetic rat kidney with interstitial macrophage accumulation (c), Unx STZ rat treated with 10 mg/kg P2X7Ri (d), and 50 mg/kg P2X7Ri-treated rat kidney (e). Glomerular macrophage abundance (f). Measurements were made at 12 weeks. *P < 0.05 UNx STZ groups vs. Sham or UNx Veh NS; not significant. c–f × 200 mag.

Renal macrophage infiltration was assessed by CD68 immunolocalization and quantified separately in glomerular and tubulointerstitial compartments (Fig. 6). Interstitial macrophage number was increased ~ 50% in diabetic UNx rats compared with both sham-operated and UNx controls (P < 0.05; Fig. 6a). Importantly, treatment with the P2X7Ri reduced interstitial macrophage inhibition in diabetic UNx rats, returning macrophage numbers to baseline levels. Representative sections comparing sham kidneys (Fig. 6b), in which there were minimal numbers of macrophages, and UNx STZ Veh kidneys (Fig. 6c) showed interstitial macrophage accumulation in the UNx diabetic kidneys. P2X7Ri at 10 m/kg (Fig. 6d) was not sufficient to reduce macrophage accumulation, however, 50 mg/kg was sufficient (Fig. 6e). In contrast to the interstitial compartment, glomerular macrophage number was not increased in diabetic rats compared with controls, and was similar across all groups (Fig. 6f).

## 4 Discussion

It is increasingly evident that P2X7R contributes to chronic inflammatory disorders: P2X7R activation promotes formation of the cryopyrin (NLRP3) inflammasome and the release of cytokines (Ferrari et al., 2006, Lister et al., 2007, Solle et al., 2001). P2X7R expression is increased in models of hypertension and diabetes (Ji et al., 2012a, Ji et al., 2012b, Menzies et al., 2015b, Vonend et al., 2004), both major risk factors for CKD in humans. Deletion or blockade of P2X7R protects against antibody-induced glomerular injury (Taylor et al., 2009, Turner et al., 2007) and deoxycorticosterone acetate salt-induced fibrosis (Ji et al., 2012b). Constitutively high renal P2X7R expression, impairs the pressure-natriuresis response and enhances susceptibility to renal vascular injury in rats (Menzies et al., 2013). Acute P2X7R antagonism improves renal blood flow, medullary perfusion and oxygenation in angiotensin-II-induced hypertension (Menzies et al., 2015a).

In the present study we found high P2X7R expression in renal biopsy samples from patients with diabetic nephropathy. This is consistent with detection of P2X7R in nephrectomy tissue from patients with type 2 diabetes and renal cell carcinoma (Solini et al., 2013). Glomerular expression of P2X7R was prevalent at ~ 50% in diabetes but was not observed in TBM disease controls. P2X7R-deficient mice were protected from the STZ-induced glomerular damage observed in WT controls. This suggests that many short-term preclinical models of increased glomerular P2X7R expression likely reflect early pro-inflammatory injury found in human glomerulosclerosis. As such, P2X7R activation in the renal vasculature or mesangium under hyperglycemic (Kreft et al., 2016), or hypertensive (Conway et al., 2012, Menzies et al., 2015a) conditions may also directly impair renal function in diabetic nephropathy. Moreover, progression of diabetic nephropathy requires coordination of glomerular and interstitial compartments (Bohle et al., 1991), typically from a combination of hyperglycemia and hypertension (Conway et al., 2012). Indeed, more severe CKD and interstitial fibrosis were observed in biopsies in which there was high interstitial P2X7R expression and interstitial fibrosis is typically found in more advanced disease (Liu, 2011).

Our observations in patients and *in vitro* studies in pHMCs suggest that activation of mesangial P2X7R may enhance early glomerular macrophage recruitment leading to injury. MCP-1 attracts macrophages that promote pro-inflammatory processes (Conti and DiGioacchino, 2001, Rovin et al., 1994). We have previously demonstrated that macrophage accumulation is reduced in P2X7R-deficient mice, as are urinary MCP-1 levels (Taylor et al., 2009). Although macrophages can transition between pro- and anti- inflammatory phenotypes, depending on the local microenvironment (Martinez and Gordon, 2014), P2X7R preferentially activates M1 polarized pro-inflammatory macrophages (de Torre-Minguela et al., 2016); therefore, P2X7R activation promotes the accumulation of deleterious pro-inflammatory macrophages.

Preclinical models of P2X7R-deficiency do not fully recapitulate the clinical reality found in patients. However, the P2X7R-deficient mouse model did allow us to demonstrate that lack of canonical P2X7R does not prevent STZ-induced hyperglycemia. Instead, P2X7R-deficiency appears to protect the kidney since these mice had reduced macrophage accrual and collagen IV deposition indicating, protection against expansion of extracellular matrix, a hallmark of fibrosis, in WT mice.

The P2X7Ri had significant effectiveness in reducing renal macrophage accrual following the establishment of diabetes. Reduction of renal interstitial macrophages with a P2X7Ri may be therapeutically beneficial in the longer-term in human diabetic nephropathy, since we identified that these patients have more severe CKD and fibrosis. An important limitation of this model was the lack of more severe glomerular and tubulointerstitial damage seen in human diabetic nephropathy. Indeed, glomerular macrophage accrual was never increased in the rat model. This may be unsurprising however, since many preclinical kidney disease models fail to capture the severity of human disease (Mullins et al., 2016). The lack of impact of P2X7R blockade on albumin excretion is consistent with the experimental findings in the P2X7R-deficient mice and suggests that P2X7R does have an important mechanistic role in the early development of glomerular damage and albuminuria; although this is despite apparent upregulation of P2X7R in the glomeruli at 12 weeks in both mice and rats with STZ-induced diabetes. Collectively, this study demonstrates the potential for selective P2X7Ri to reduce renal macrophage accumulation, supporting future investigations of P2X7R inhibition in diabetes and other renal diseases characterized by macrophage infiltration.

Prophylactic use of a P2X7Ri has proven reno-protective in multiple kidney disease models (Menzies et al., 2016), but it is unclear whether blockade can reverse an established fibrotic phenotype. The data presented here suggest that P2X7Ri may have specific anti-inflammatory and anti-macrophage role in diabetic nephropathy. Further longitudinal studies are required to determine whether P2X7Ri can offer real clinical benefit to early, and more established fibrotic kidney disease in diabetes, as well as other forms of human CKD.

## Funding Sources

RIM is a BHF Immediate Postdoctoral Basic Science Research Fellow (Award number FS/15/60/31510). JWRB received a MRC/Kidney Research UK Joint Clinical Training Fellowship (Award number G0901956). FWKT is supported by the Diamond Fund from Imperial College Healthcare Charity and Ken and Mary Minton Chair of Renal Medicine. Part of this work was supported by the National Institute for Health Research (NIHR) Biomedical Research Centre, Imperial College Healthcare NHS Trust and Imperial College London. The funding bodies did not dictate the study design, data collection, data analysis, interpretation, or writing of the report.

## Conflicts of Interest

FWKT has received research project grants from AstraZeneca Limited, Baxter Biosciences, GSK, MedImmune, Rigel Pharmaceuticals and Roche Palo Alto, and has consultancy agreements with MedImmune and Rigel Pharmaceuticals. RJU is presently Chief Scientist (secondment) at AstraZeneca CVMD iMed R&D, Sweden. JTN has received grants from AstraZeneca and UCB. MAB has received grants from AstraZeneca.

## Author Contributions

RIM analyzed the data, produced the figures, and wrote the manuscript. JWRB performed the experiments and collected the data. JTN, FWKT and RJU supervised design and data analysis. JTN, FWKT, JJM, MAB and RJU provided critical interpretation of study results that helped RIM produce a comprehensive report. All authors contributed through critical reading and feedback that refined the manuscript to completion.

## Appendix A Supplementary data

Supplementary material Image 1

Suppl. Fig 1. Immunohistochemistry of P2X7R in diabetic nephropathy. Typical appearance of tubules in diabetic nephropathy (a). Glomerular P2X7R expression in diabetic nephropathy (b). P2X7R-positive interstitial cells in diabetic nephropathy (c). Biopsy tissue probed with P2X7R antibody after prior incubation with specific immunizing peptide in 1:1 ratio (d). a-d x200 mag.

Suppl. Fig 2. Immunohistochemistry for P2X7R in Thin Basement Membrane disease and Lupus Nephritis Typical appearance of a glomerulus from a patient with TBM lesion, with no appreciable P2X7R staining (a). Tubular appearance in TBM disease (b). Glomerulus from a patient with class IV proliferative lupus nephritis (c). a-c x200 mag.

Suppl. Fig 3. P2X7 inhibitor (A438079) reduced MCP-1 release from high concentration glucose and BzATP stimulated mesangial cells. No apparent dose-dependence was seen over a range of concentrations of A438079 from 5 - 50 μM, with all proving equally effective (all comparisons to 30mM D-glucose (D) group). ** P < 0.01, ***P < 0.001

Suppl. Fig. 4. Assessment of A438079 cytotoxicity using MTS assay. A438079 had no effect on cell viability over the 5 -50μM concentration range. Normoglycemic RPMI 1640 medium was supplemented with the appropriate concentrations of inhibitor or drug vehicle and cultures incubated at 37^o^C for 48 hours. D: D-glucose. NS: Not significant.

Suppl. Fig. 5. Urinary Creatinine levels in diabetic mice (a) Serum creatinine at week 12. Creatinine was elevated in both diabetic groups compared to controls, but was equivalent in WT and P2X7R-deficient diabetic mice. (b) uACR at week 12. uACR was elevated in both diabetic groups compared to controls, but failed to reach statistical signficance. A trend towards higher uACR values was seen in STZ-treated P2X7R-deficient mice compared to WT, but with a wide standard deviation of values in the former group. *** P < 0.001, NS: Not significant.

## References

[bb0005] Afkarian M., Sachs M.C., Kestenbaum B., Hirsch I.B., Tuttle K.R., Himmelfarb J., de Boer I.H. (2013). Kidney disease and increased mortality risk in type 2 diabetes. J. Am. Soc. Nephrol..

[bb0010] Bohle A., Wehrmann M., Bogenschutz O., Batz C., Muller C.A., Muller G.A. (1991). The pathogenesis of chronic renal failure in diabetic nephropathy. Investigation of 488 cases of diabetic glomerulosclerosis. Pathol. Res. Pract..

[bb0015] Broom D.C., Matson D.J., Bradshaw E., Buck M.E., Meade R., Coombs S., Matchett M., Ford K.K., Yu W., Yuan J., Sun S.H., Ochoa R., Krause J.E., Wustrow D.J., Cortright D.N. (2008). Characterization of N-(Adamantan-1-ylmethyl)-5-[(3R-aminopyrrolidin-1-yl)methyl]-2-chloro-benzamide, a P2X7 antagonist in animal models of pain and inflammation. J. Pharmacol. Exp. Ther..

[bb0020] Conti P., DiGioacchino M. (2001). MCP-1 and RANTES are mediators of acute and chronic inflammation. Allergy Asthma Proc..

[bb0025] Conway B.R., Rennie J., Bailey M.A., Dunbar D.R., Manning J.R., Bellamy C.O., Hughes J., Mullins J.J. (2012). Hyperglycemia and renin-dependent hypertension synergize to model diabetic nephropathy. J. Am. Soc. Nephrol..

[bb0030] Costa-Junior H.M., Vieira F.S., Coutinho-Silva R. (2011). C terminus of the P2X7 receptor: treasure hunting. Purinergic Signal.

[bb0035] Coutinho-Silva R., Robson T., Beales P.E., Burnstock G. (2007). Changes in expression of P2X7 receptors in NOD mouse pancreas during the development of diabetes. Autoimmunity.

[bb0040] Ferrari D., Pizzirani C., Adinolfi E., Lemoli R.M., Curti A., Idzko M., Panther E., Di Virgilio F. (2006). The P2X7 receptor: a key player in IL-1 processing and release. J. Immunol..

[bb0045] Gómez-Guerrero C., Hernández-Vargas P., López-Franco O., Ortiz-Muñoz G., Egido J. (2005). Mesangial cells and glomerular inflammation: from the pathogenesis to novel therapeutic approaches. Curr. Drug Targets. Inflamm. Allergy.

[bb0050] Groop P.-H., Thomas M.C., Moran J.L., Wadèn J., Thorn L.M., Mäkinen V.-P., Rosengård-Bärlund M., Saraheimo M., Hietala K., Heikkilä O., Forsblom C. (2009). The presence and severity of chronic kidney disease predicts all-cause mortality in type 1 diabetes. Diabetes.

[bb0055] Gross J.L., De Azevedo M.J., Silveiro S.P., Canani L.H., Caramori M.L., Zelmanovitz T. (2005). Diabetic nephropathy: diagnosis, prevention, and treatment. Diabetes Care.

[bb0060] Ihm C.G., Park J.K., Hong S.P., Lee T.W., Cho B.S., Kim M.J., Cha D.R., Ha H. (1998). A high glucose concentration stimulates the expression of monocyte chemotactic peptide 1 in human mesangial cells. Nephron.

[bb0065] Ji X., Naito Y., Hirokawa G., Weng H., Hiura Y., Takahashi R., Iwai N. (2012). P2X(7) receptor antagonism attenuates the hypertension and renal injury in Dahl salt-sensitive rats. Hypertens. Res..

[bb0070] Ji X., Naito Y., Weng H., Endo K., Ma X., Iwai N. (2012). P2X7 deficiency attenuates hypertension and renal injury in deoxycorticosterone acetate-salt hypertension. Am. J. Physiol. Ren. Physiol..

[bb0075] Ke H.Z., Qi H., Weidema A.F., Zhang Q., Panupinthu N., Crawford D.T., Grasser W.A., Paralkar V.M., Li M., Audoly L.P., Gabel C.A., Jee W.S.S., Dixon S.J., Sims S.M., Thompson D.D. (2003). Deletion of the P2X7 nucleotide receptor reveals its regulatory roles in bone formation and resorption. Mol. Endocrinol..

[bb0080] Kreft E., Kowalski R., Jankowski M., Szczepańska-Konkel M. (2016). Renal vasculature reactivity to agonist of P2X7 receptor is increased in streptozotocin-induced diabetes. Pharmacol. Rep..

[bb0085] Lister M.F., Sharkey J., Sawatzky D.A., Hodgkiss J.P., Davidson D.J., Rossi A.G., Finlayson K. (2007). The role of the purinergic P2X7 receptor in inflammation. J. Inflamm..

[bb0090] Liu Y. (2011). Cellular and molecular mechanisms of renal fibrosis. Nat. Rev. Nephrol..

[bb0095] Martinez F.O., Gordon S. (2014). The M1 and M2 paradigm of macrophage activation: time for reassessment. F1000Prime Rep..

[bb0100] Mason R.M., Wahab N.A. (2003). Extracellular matrix metabolism in diabetic nephropathy. J. Am. Soc. Nephrol..

[bb0105] Menzies R.I., Unwin R.J., Dash R.K., Beard D.A., Cowley A.W., Carlson B.E., Mullins J.J., Bailey M.A. (2013). Effect of P2X4 and P2X7 receptor antagonism on the pressure diuresis relationship in rats. Front. Physiol..

[bb0110] Menzies R.I., Howarth A., Unwin R., Tam F., Mullins J., MA B. (2015). Inhibition of the purinergic P2X7 receptor improves renal perfusion in angiotensin-II infused rats. Kidney Int..

[bb0115] Menzies R.I., Unwin R.J., Bailey M.A. (2015). Renal P2 receptors and hypertension. Acta Physiol..

[bb0120] Menzies R.I., Tam F.W., Unwin R.J., Bailey M.A. (2016). Purinergic signaling in kidney disease. Kidney Int..

[bb0125] Mullins L.J., Conway B.R., Menzies R.I., Denby L., Mullins J.J. (2016). Renal disease pathophysiology and treatment: contributions from the rat. Dis. Model. Mech..

[bb0130] Nicke A., Kuan Y.-H., Masin M., Rettinger J., Marquez-Klaka B., Bender O., Górecki D.C., Murrell-Lagnado R.D., Soto F. (2009). A functional P2X7 splice variant with an alternative transmembrane domain 1 escapes gene inactivation in P2X7 knock-out mice. J. Biol. Chem..

[bb0135] Parving H.H., Andersen A.R., Smidt U.M., Svendsen P.A. (1983). Early aggressive antihypertensive treatment reduces rate of decline in kidney function in diabetic nephropathy. Lancet.

[bb0140] Rovin B.H., Rumancik M., Tan L., Dickerson J. (1994). Glomerular expression of monocyte chemoattractant protein-1 in experimental and human glomerulonephritis. Lab. Invest..

[bb0145] Savige J., Gregory M., Gross O., Kashtan C., Ding J., Flinter F. (2013). Expert guidelines for the management of Alport syndrome and thin basement membrane nephropathy. J. Am. Soc. Nephrol..

[bb0150] Savill J., Smith J., Sarraf C., Ren Y.I., Abbott F., Rees A. (1992). Glomerular mesangial cells and inflammatory macrophages ingest neutrophils undergoing apoptosis. Kidney Int..

[bb0155] Shaw J.E., Sicree R.A., Zimmet P.Z. (2010). Global estimates of the prevalence of diabetes for 2010 and 2030. Diabetes Res. Clin. Pract..

[bb0160] Shieh C.-H., Heinrich A., Serchov T., van Calker D., Biber K. (2014). P2X7-dependent, but differentially regulated release of IL-6, CCL2, and TNF-α in cultured mouse microglia. Glia.

[bb0165] Solini A., Iacobini C., Ricci C., Chiozzi P., Amadio L., Pricci F., Di Mario U., Di Virgilio F., Pugliese G., Mario U. Di, Virgilio F. Di, Pugliese G. (2005). Purinergic modulation of mesangial extracellular matrix production: role in diabetic and other glomerular diseases. Kidney Int..

[bb0170] Solini A., Menini S., Rossi C., Ricci C., Santini E., Blasetti Fantauzzi C., Iacobini C., Pugliese G. (2013). The purinergic 2X7 receptor participates in renal inflammation and injury induced by high-fat diet: possible role of NLRP3 inflammasome activation. J. Pathol..

[bb0175] Solini A., Usuelli V., Fiorina P. (2014). The dark side of extracellular ATP in kidney diseases. J. Am. Soc. Nephrol..

[bb0180] Solle M., Labasi J., Perregaux D.G., Stam E., Petrushova N., Koller B.H., Griffiths R.J., Gabel C.A. (2001). Altered cytokine production in mice lacking P2X(7) receptors. J. Biol. Chem..

[bb0185] Stokes L., Surprenant A. (2007). Purinergic P2Y2 receptors induce increased MCP-1/CCL2 synthesis and release from rat alveolar and peritoneal macrophages. J. Immunol..

[bb0190] Tam F.W., Riser B.L., Meeran K., Rambow J., Pusey C.D., Frankel A.H. (2009). Urinary monocyte chemoattractant protein-1 (MCP-1) and connective tissue growth factor (CCN2) as prognostic markers for progression of diabetic nephropathy. Cytokine.

[bb0195] Taylor S.R.J., Turner C.M., Elliott J.I., McDaid J., Hewitt R., Smith J., Pickering M.C., Whitehouse D.L., Cook H.T., Burnstock G., Pusey C.D., Unwin R.J., Tam F.W.K. (2009). P2X7 deficiency attenuates renal injury in experimental glomerulonephritis. J. Am. Soc. Nephrol..

[bb0200] Tervaert T.W.C., Mooyaart A.L., Amann K., Cohen A.H., Cook H.T., Drachenberg C.B., Ferrario F., Fogo A.B., Haas M., de Heer E., Joh K., Noël L.H., Radhakrishnan J., Seshan S.V., Bajema I.M., Bruijn J.A. (2010). Pathologic classification of diabetic nephropathy. J. Am. Soc. Nephrol..

[bb0205] The Diabetes Control and Complications Trial Research Group (1993). The effect of intensive treatment of diabetes on the development and progression of long-term complications in insulin-dependent diabetes mellitus. N. Engl. J. Med..

[bb0210] de Torre-Minguela C., Barbera-Cremades M., Gomez A.I., Martin-Sanchez F., Pelegrin P. (2016). Macrophage activation and polarization modify P2X7 receptor secretome influencing the inflammatory process. Sci. Rep..

[bb0215] Turner C.M., Tam F.W.K., Lai P.-C., Tarzi R.M., Burnstock G., Pusey C.D., Cook H.T., Unwin R.J. (2007). Increased expression of the pro-apoptotic ATP-sensitive P2X7 receptor in experimental and human glomerulonephritis. Nephrol. Dial. Transplant..

[bb0220] Vonend O., Turner C.M., Chan C.M., Loesch A., Dell'Anna G.C., Srai K.S., Burnstock G., Unwin R.J. (2004). Glomerular expression of the ATP-sensitive P2X receptor in diabetic and hypertensive rat models. Kidney Int..

[bb0225] Wada J., Makino H. (2013). Inflammation and the pathogenesis of diabetic nephropathy. Clin. Sci..

[bb0230] Wada T., Furuichi K., Sakai N., Iwata Y., Yoshimoto K., Shimizu M., Takeda S.I., Takasawa K., Yoshimura M., Kida H., Kobayashi K.I., Mukaida N., Naito T., Matsushima K., Yokoyama H. (2000). Up-regulation of monocyte chemoattractant protein-1 in tubulointerstitial lesions of human diabetic nephropathy. Kidney Int..

[bb0235] Zeisberg M., Neilson E.G. (2010). Mechanisms of tubulointerstitial fibrosis. J. Am. Soc. Nephrol..

