# Supplementary figures and images for: Hyperglycemia-induced Renal P2X7 Receptor Activation Enhances Diabetes-related Injury
Source: eBioMedicine. 2017 Apr 20;19:73–83. doi: 10.1016/j.ebiom.2017.04.011 (PMC5440600; doi:10.1016/j.ebiom.2017.04.011)

## Slide 1
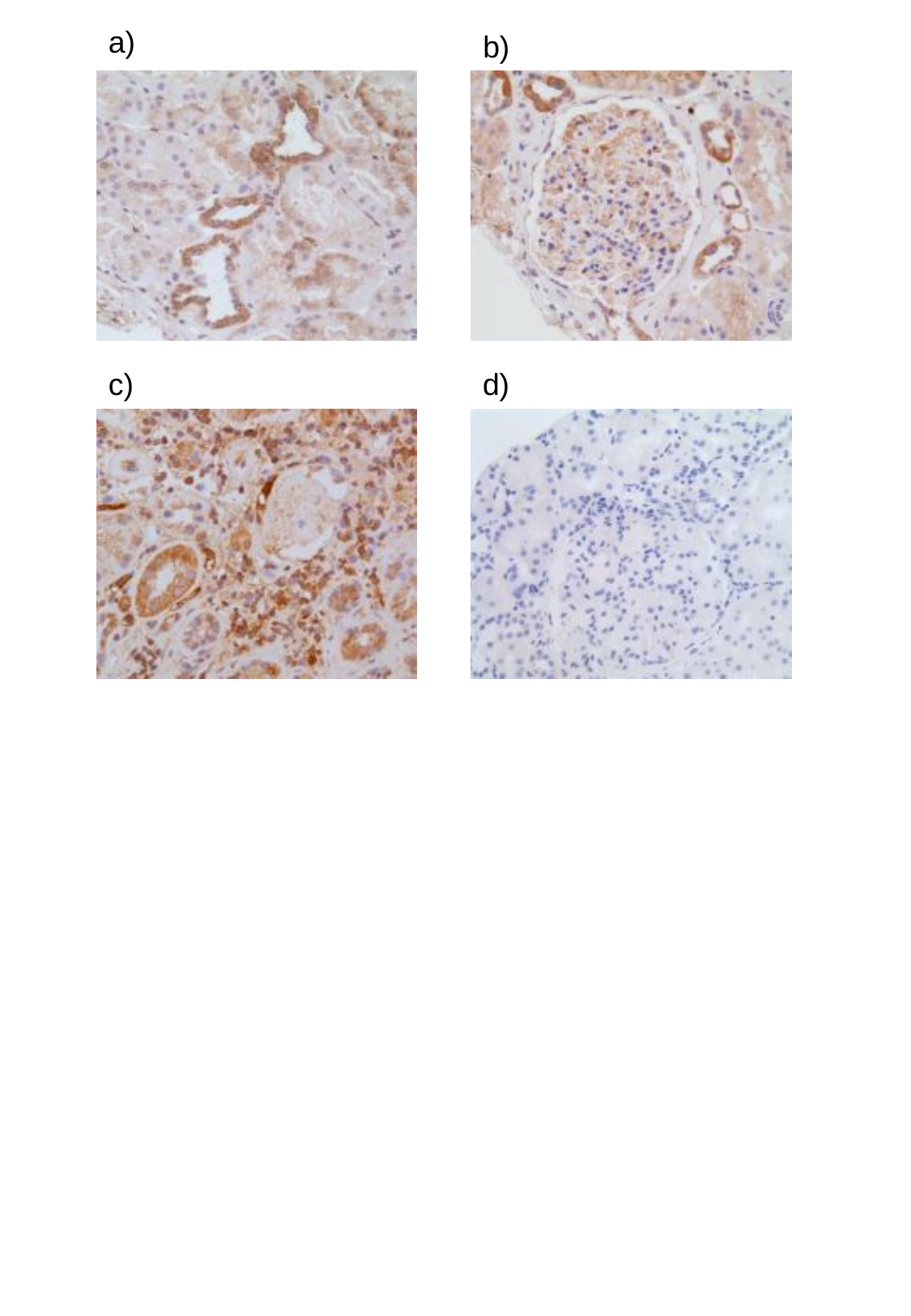

a)
b)
c)
d)

## Slide 2
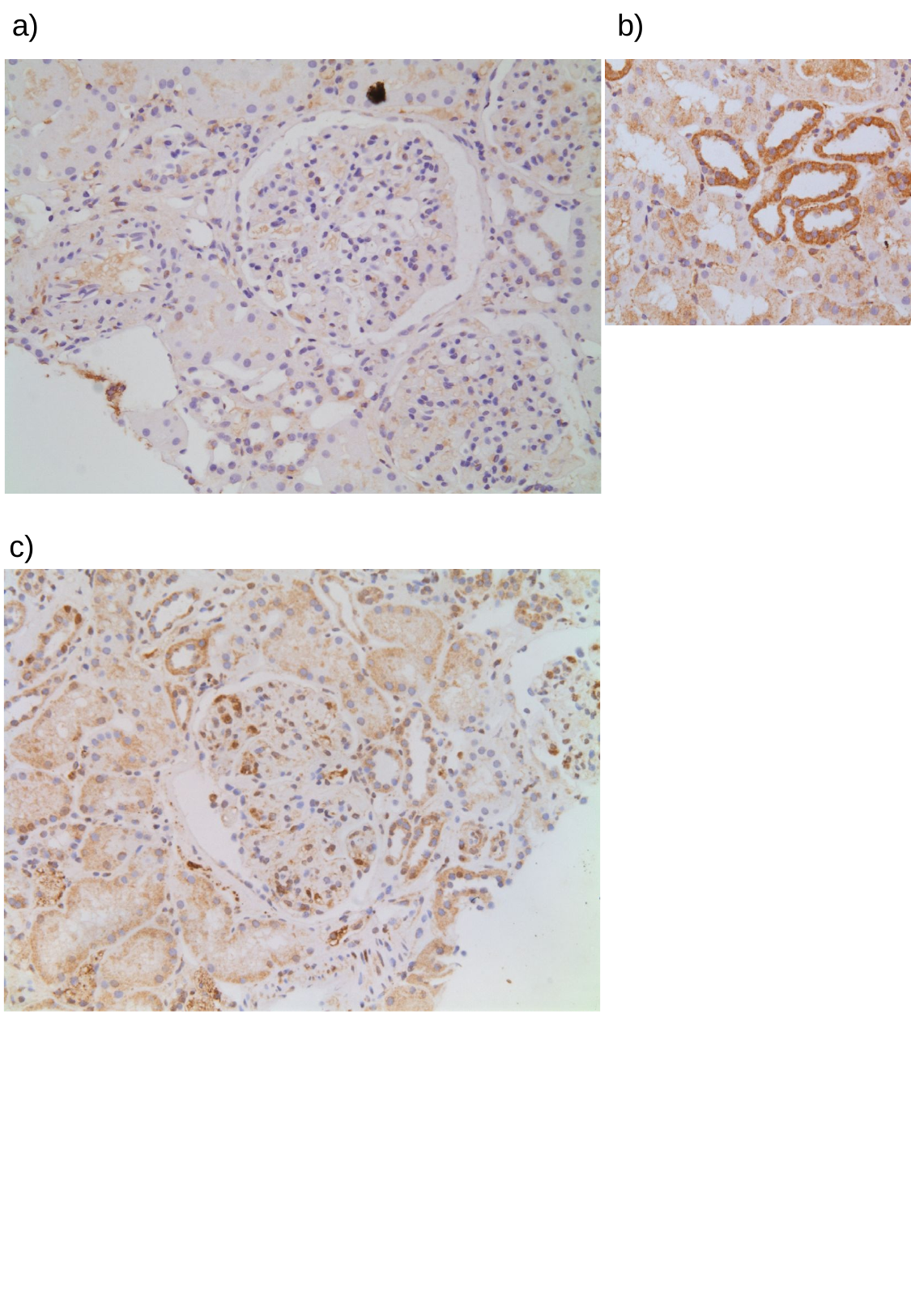

a)
b)
c)

## Slide 3
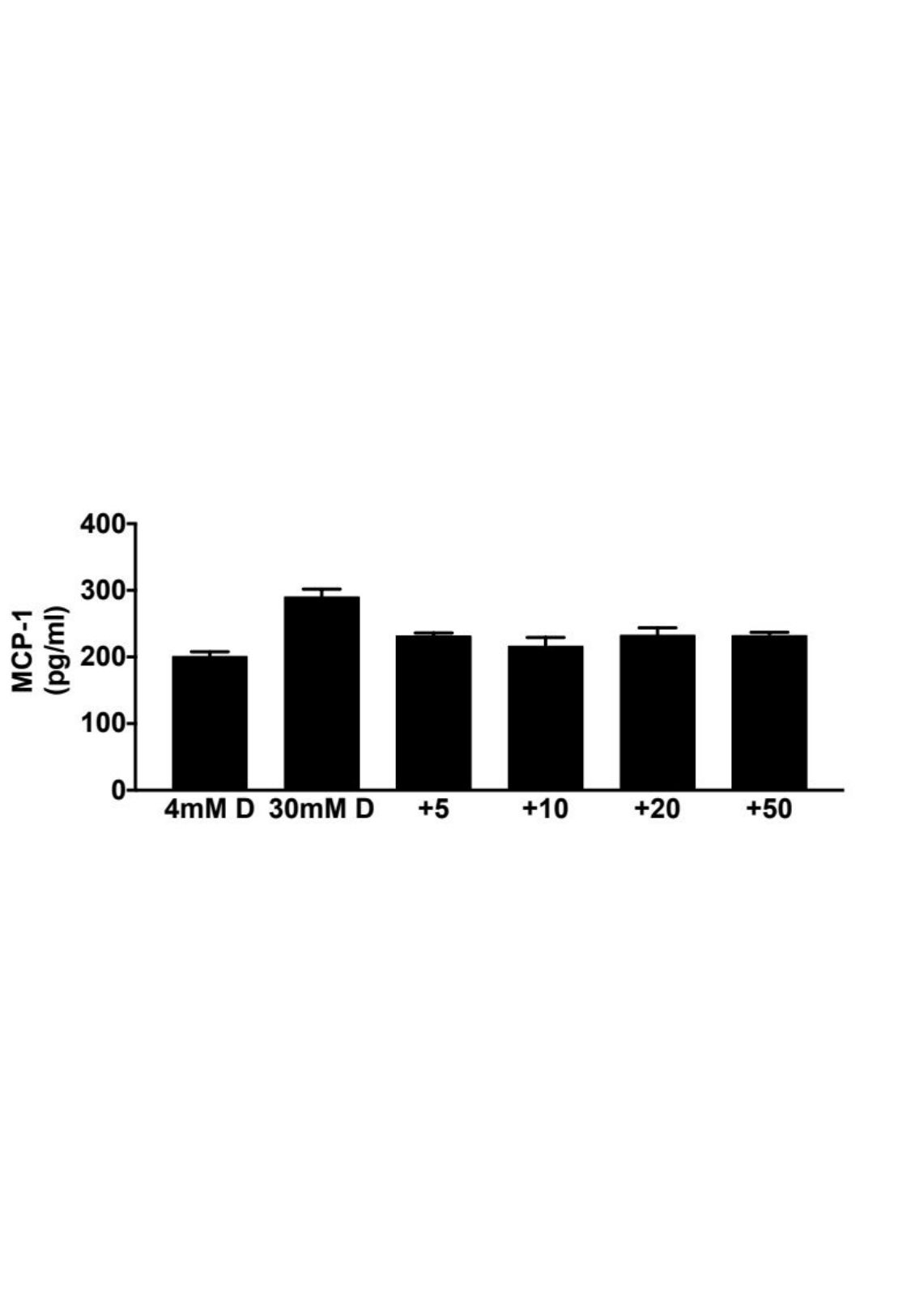

## Slide 4
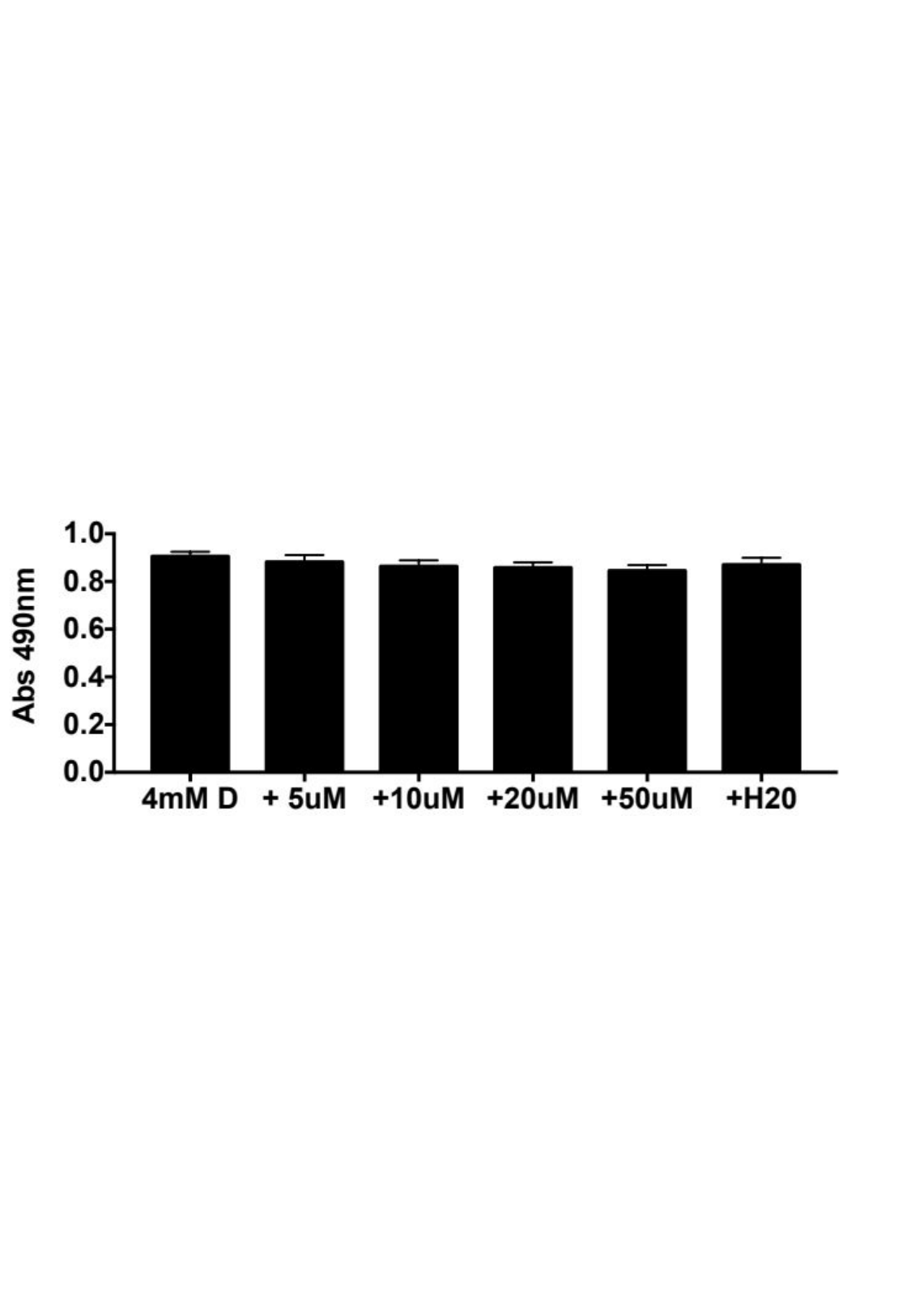

## Slide 5
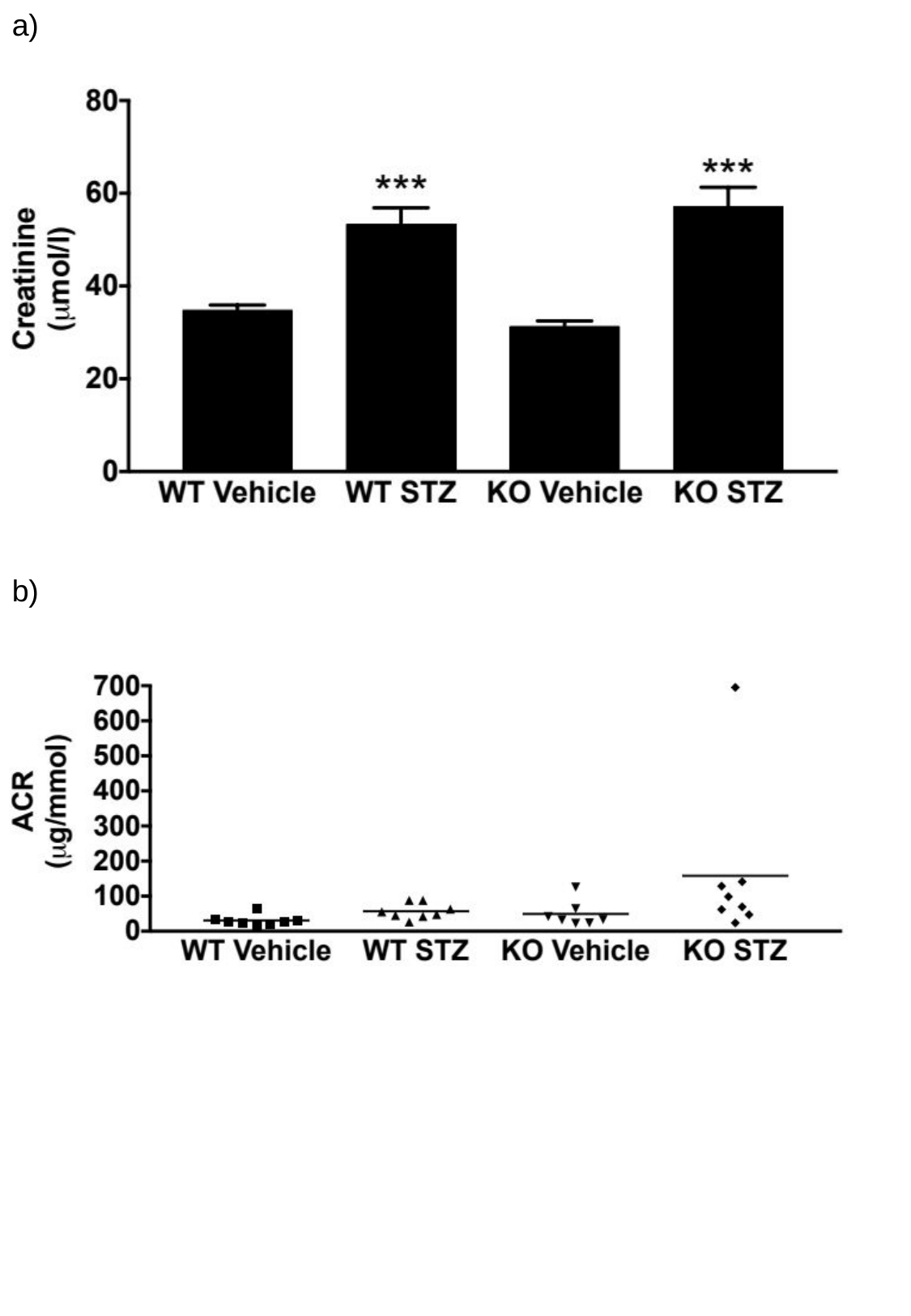

a)
b)

Supplement: Supplementary file 1 — Supplementary material [file mmc1.pptx]
